# Validation of a method of broth microdilution for the determination of antibacterial activity of essential oils

**DOI:** 10.1186/s13104-021-05838-8

**Published:** 2021-12-02

**Authors:** David Vanegas, Andrea Abril-Novillo, Aleksandr Khachatryan, Lourdes Jerves-Andrade, Eugenia Peñaherrera, Nancy Cuzco, Isabel Wilches, Jessica Calle, Fabián León-Tamariz

**Affiliations:** Deparment of Biosciences, Group of Medicinal Plants and Natural Products, Faculty of Chemistry, School of Biochemistry and Pharmacy, Universidad de Cuenca, Cuenca, Ecuador

**Keywords:** Essential oils, *Escherichiacoli*, Microdilution assay, Validation, Sensitivity, Specificity

## Abstract

**Objective:**

The aim of the present study was to adapt and optimize a broth microdilution method and compare it to the agar dilution method for the evaluation of activity of essential oils from medicinal plants against Gram-negative bacteria. Based on bibliographic research, active and not active oils were selected. The sensitivity and specificity were established as parameters for validation. The comparison between both methods was made using contingency analysis tables, based on the observed frequencies. For both methods, the minimum inhibitory concentration was determined against *Escherichia* *coli* strains, in an essential oil concentration range between 0.03 and 0.48% (v/v).

**Results:**

A stable emulsion formation was achieved with the addition of Tween 80 and constant agitation, guaranteeing the continuous contact of oil with bacteria (critical step in the microdilution method). The statistical analysis of results obtained with both methods presented a good sensitivity and specificity (100% in both cases), which let us correctly discriminate between active and non-active oils. The values obtained for the minimal inhibitory concentration were independent of the technique used. Finally, the obtained results show that the validated microtechnique allows important diminishment of time and resources for investigations dealing with essential oils or lipophilic extracts evaluation.

**Supplementary Information:**

The online version contains supplementary material available at 10.1186/s13104-021-05838-8.

## Introduction

The resistance to antimicrobials is a global health problem, probably related to millions of deaths each year [1, 2]. Natural products have become an option for scientists in the search of new treatments to confront resistant and multidrug-resistant bacteria (MDR). Plant products include a range of structurally complex and diverse metabolites [3]. One important group corresponds to the essential oils, which are volatile compounds found in certain families at different structures [3]. Essential oils have several applications in food and cosmetic industries, but also, important biological properties such as their antibacterial activity against many pathogenic microorganisms, which can be an alternative to fight resistant bacteria [3–5].

Nowadays, there are different “in vitro” methods to evaluate the antibacterial activity of natural compounds, being agar and broth dilution methods the gold standard to determine the minimum inhibitory concentration (MIC) values in a more exact approach [6]. However, in many of the articles published, results do not coincide among them due to the use of different methods, possibly standardized in each laboratory, which are not internationally certified; in addition, most of them employ techniques of poor sensitivity to determine antibacterial activity for essential oils, making it difficult and delaying cross-publication comparisons of new findings and advances [7–9]. Many factors might influence the sensitivity of the different methods used, such as concentration of inoculum, interactions between the components of the essential oil and culture medium, formation of emulsion, volatility of some metabolites, concentration of surfactants, time and temperature of incubation, among others [7, 8]. Therefore, it is convenient to define the conditions under which the experiments were carried out in order to control them.

The main objective of this study was to validate a broth microdilution standard method by comparing it against the agar dilution method in order to determine the MIC of selected essential oils, while achieving high sensitivity and specificity.

## Main text

### Sample size

The number of samples were defined using the equation for binary data; through the calculation of sensitivity (n_1_) and specificity (n_2_), the result established 16 essential oil samples to each parameter, corresponding to different plants. Total sample size, including prevalence, was n  = 32 essential oils. To assure that the minimum sample size was fulfilled, the final sample size was determined to be 33 essential oils to correctly validate the microdilution method.

Given the fact that sensitivity is represented by the admissible error (Zα/2), the standardized value of the normal distribution corresponds to a confidence level of 100% (1 − α).

### Essential oils selection

Thirty-three medicinal plants were selected based on literature review, where the activity of the respective essential oil against *E.* *coli* were reported. Limits for activity were established at 0.03 to 0.48% v/v, based on the reported MIC and cytotoxicity for the different oils [3, 4, 10–33]. The list of selected species are presented in Tables 1 (purchased at herbal shop), and 2 (plants at local markets). Essential oils were obtained by hydrodistillation method where fresh plant material (100 g) was dried, cut, and placed with distilled water (1 L) into a Clevenger`s type apparatus for 3 h. The mix of oil and water was collected and dried over anhydrous sodium sulfate. After the centrifugation process (3000 rpm for 5 min), supernatant (essential oils) was stored in amber vials at − 20 °C until analysis.

**Table 1:** Essential oils of therapeutic grade tested and references of their activity or no activity against *E.* *coli* strains

Essential oil	Common name	Part used	Reported antibacterial activity MIC^a^	References
*Eucalyptus* *globulus*	Blue gum	LT	> 0.5% v/v	[10]
*Syzygium* *aromaticum*	Clove	BD	0.24% v/v	[10]
*Cinnamomum* *cassi*	Chinese cinnamon	W	0.1% v/v	[11]
*Boswellia* *serrata*	Sallaki	R	9.25% v/v	[12]
*Lavandula* *angustifolia*	Lavender	FL	1.07% v/v	[13]
*Cymbopogon* *flexuous*	Cochin grass	LT	> 0.08% v/v	[14]
*Mentha* × *piperita*	Peppermint	LT	0.004% v/v	[15]
*Rosmarinus* *officinalis*	Rosemary	LT	0.014% v/v	[4]
*Mentha* *spicata*	Spearmint	LT	0.04% v/v	[15]
*Melaleuca* *alternifolia*	Tea tree	LT	> 1% v/v	[16]
*Citrus* *racemose*	Grapefruit	P	0.63% v/v	[17]
*Citrus* *limonum*	Lemon	P	1% v/v	[18]
*Citrus* *aurantifolia*	Key lime	P	1% v/v	[19]
*Citrus* *sinensis*	Sweet orange	P	0.08% v/v	[20]
*Pogostemon* *cablin*	Patchouli	NN	0.05% v/v	[16]
*Salvia* *sclarea*	Clary sage	NN	> 53.2% v/v	[21]
*Citrus* *bergamia*	Bergamot orange	NN	1% v/v	[18]

*BD* bud; *FL* flower; *LT* leaves; *P* peel; *NN* not named; *R* resin; *W* wood

^a^The range of activity/no-activity were determined based on reported MIC and the cytotoxicity of essential oils

**Table 2:** Essential oils of medicinal plants from local markets, and references of their activity or no activity against *E.* *coli* strains

Plants	Common name	Plant part	Reported antibacterial activity MIC^a^	References
*Zingiber* *officinale*	Ginger	RH	> 2% v/v	[19]
*Foeniculum* *vulgare*	Fennel	S	0.007 %v/v	[4]
*Pimpinella* *anisum*	Anise	S	0.10% v/v	[22]
*Coriandrum* *sativum*	Coriander	S	0.07% v/v	[23]
*Curcuma* *longa*	Turmeric	RH	48.02% v/v	[24]
*Piper* *nigrum*	Black pepper	FR	> 2% v/v	[19]
*Peumus* *boldus*	Boldo	LT (dry)	0.003% v/v	[4]
*Laurus* *nobilis*	Baby laurel	LT (dry)	1.25% v/v	[25]
*Petroselinum* *crispum*	Parsley	S	1.08% v/v	[3]
*Elettaria* *cardamomum*	Green cardamom	S	1% v/v	[26]
*Ocotea* *quixos*	Ishpingo	S	1.68% v/v	[27]
*Eucalyptus* *citriodora*	Eucalyptus	LT	0.2% v/v	[21]
*Salvia* *microphylla*	Baby sage	LT	> 53.2% v/v	[28]
*Cuminum* *cyminum*	Cumin	S	0.001% v/v	[29]
*Illicium* *verum*	Star anise	S	0.001% v/v	[30]
*Pimenta* *dioica*	All spice	FR	0.19% v/v	[22]

*FR* fruit; *L* *T* leaves; *RH* rhizome; *S* seed

^a^The range of activity/no-activity were determined based on reported MIC and the cytotoxicity of essential oils

### Bacterial strain

*Escherichia* *coli* (ATCC 25922) standard strains were used and cryo-conserved at concentrations of 5 × 10^4^ − 5 × 10^5^ CFU/mL using 10% glycerol as cryoprotectant and stored at − 80 °C [34, 35].

### Preliminary assays—dilution in agar

Agar dilution method was carried out with tempered agar (45 °C), essential oil and Tween 20 (0.5%) were mixed to obtain an emulsion; 25 mL of this emulsion was poured into petri dishes to obtain a 3 to 4 mm depth [6, 36]. After solidification of the culture medium (MHA, Merck, Germany), eight spots with 1.5 µL each one (different dilutions) were scattered using a micropipette and were allowed to dry (each spot containing 1 × 10^4^ CFU/mL *E.* *coli*), and incubated as inverted dishes at 35  ±  2 °C for 16–20 h [6]. Procedure was performed in triplicate, using inoculum as positive control and culture medium as negative control; ampicillin (Ampibex^®^ 1 g/4 mL, Life, Ecuador) was used as antibiotic control range from 16 to 1 µg/mL [31]. Minimum inhibitory concentration was determined based on the absence of visual turbidity after 20 h of incubation [6].

### Microdilution in broth

Microdilution test was performed as established in CLSI standard methods with the following modifications: first, the Tryptic Soy Broth medium (Becton Dickinson and Company, USA) was supplemented with Tween 80 at a final concentration of 0.5% [37], 30 min sonication and vortex homogenization for 8 min were used to obtain a stable essential oil emulsion [38]. The serial dilutions of the essential oil in broth were prepared in micro tubes of 1 mL with a concentration range from 0.06 to 0.96% (v/v). Then, 100 µL of each dilution were transferred into a 96-well microplate in 2 × 11 columns. Bacterial suspension (100 µL) was inoculated in each well with 1 × 10^5^ CFU/mL of *E.* *coli* ATCC 25922 to obtain final concentrations of 5 × 10^4^ CFU/mL and a final volume of 200 µL per well. The inoculum (positive control) and culture medium (negative control) were put into the first column of the microplate, and the ampicillin antibiotic control ranging from 64 to 0.5 µg/mL in the final column. Finally, microplate was incubated with a sterile film cover for 18–24 h at 35 ± 2 °C [6, 31] Subsequently, 20 µL of bacterial growth indicator, resazurin, was added to wells, which were then incubated for 30 min at 35 ± 2 °C. The lowest concentration of essential oil that visually showed no growth was determined as MIC [3].

### Validation of a standardized microdilution method

For the validation of the proposed method, sensitivity and specificity were determined based on the results obtained from the analysis of essential oils against the bacterial strains through the comparison of both methods. Given that the test works with a binomial variable: active (1) and non-active (0), a comparison between both methods was carried out using 2 × 2 contingency analysis tables. Based on the observed frequencies (Active-Active, Active-Non active, Non active-Active and Non active-Non active), sensitivity and specificity were calculated through Eqs. 1, 2:

1 $$ Sensibility = \frac{{O_{AA} }}{{O_{AA} + O_{NA} }} $$

2 $$ Specificity = \frac{{O_{NN} }}{{O_{AN} + O_{NN} }} $$

where O_AA_ represents true positive values, O_NA_ false positive values, O_NN_ true negative values and O_AN_ true false negative values.

To establish if there was independence between the results of essential oil antimicrobial activity and the values of MIC in both assays, a Chi-Square Test and Fisher’s exact F Test were performed, using a 2 × 2 contingency table analysis. Based on the results, the *p* value was calculated for both tests, with the purpose of accepting or rejecting the null hypothesis, which determines the independence of data. The independence of the MIC values was determined only with positive results with a MIC  ≤ 0.48% (v/v).

Finally, to determine if there was independence between the antibacterial activity determination method and the MIC, Chi-square Test with Yates correction, Chi-square with 2000 Monte Carlo simulation and Fisher’s exact test were performed.

### Results and discussion

Thirty-three essential oils from medicinal plants were evaluated for their antibacterial activity against *E.* *coli* ATCC 25922; fifteen of them were active inside the study range. These include *E.* *citriodora,* *Z.* *officinale,* *S.aromaticum,* *C.* *cassia,* *L.* *angustifolia,* *C.* *flexuosus* *M.* × *piperita,* *M.* *spicata,* *M.* *altinifolial,* *C.* *cyminum,* *P.* *dioica,* *C.* *sativum,* *P.* *boldus,* *L.* *nobilis* and *O.* *quixos*. Eighteen did not result active in a maximum concentration of 0.48% (v/v) in both methods. With results of the activity of the essential oils against *E.* *coli* ATCC 25922*,* sensitivity and specificity were determined with a value of 100%, being highly sensitive and highly specific. The number of trials considering the replicas was 156 tests for microdilution and 159 tests for dilution method.

The *p* values of the Chi-Square Test and Fisher’s Exact F of the frequency table of activity, showed a value of 1, indicating independence between the applied methods and their respective results regarding the activity or non-activity of the essential essentials. This means that the activity in both cases does not depend on the method applied (both following the CLSI guidelines).

For the control of methodology, specific *E.* *coli* ATCC 25922 strains were considered with MIC values  ≤ 8 µg/mL for ampicillin as a reference, which was used as control for each assay plate. The concentration of the bacterial inoculum is an important factor to consider due to its relation with an increase or decrease of the MIC, generating false positive or negative results as pointed in official method M07-A10 and by other authors [39]. Besides, lipophilicity of essential oils might represents another limitation for the methodology, avoiding the normal contact of bacteria with natural metabolites. The use of emulsification agents such as Tween 80 in broth and Tween 20 in agar, with concentrations of 0.5% (v/v) in both cases, resulted in a good dispersion of oils in a liquid medium [40, 41]. Sonication was applied to the emulsion for 30 min showing the reduction of droplet size and the increment of the emulsion’s stability as well as their antimicrobial activity [42]. To guarantee contact of the oil and the microorganism all time, constant agitation was employed during the incubation period.

**Fig. 1:**
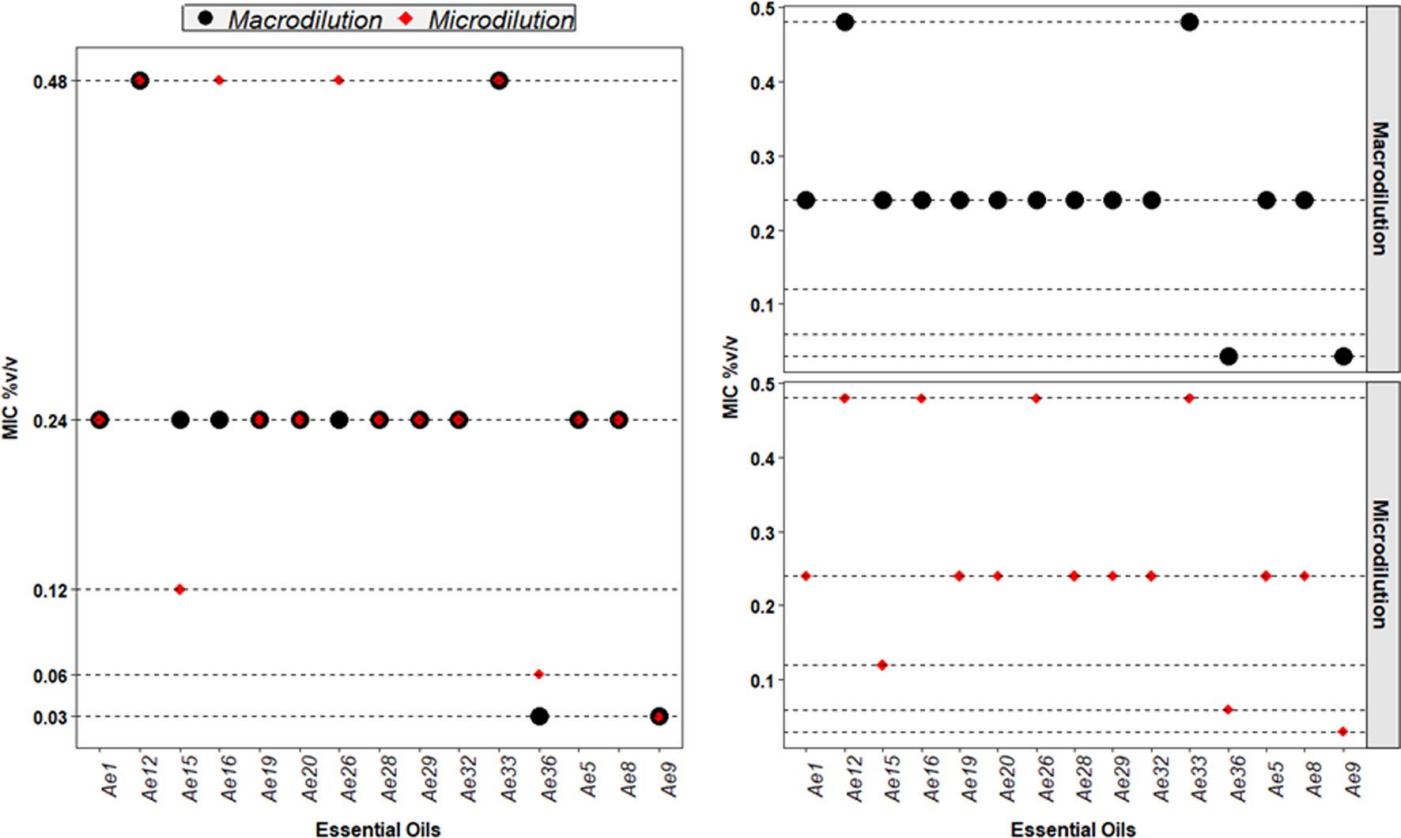
Comparison of the antimicrobial activity of the essential oils at a MIC  ≤  0.48% (v/v) for the micro and macro dilution methods of *E.* *citriodora* (*Ae1*), *Z.* *officinale* (*Ae5*), *S.* *aromaticum* (*Ae8*), *C.* *cassia* (*Ae9*), *L.* *angustifolia* (*Ae12*), *C.* *flexuosus* (*Ae15*), *M.* × *piperita* (*Ae16*), *M.* *spicata* (*Ae19*), *M.* *altinifolial* (*Ae20*), *C.* *cyminum* (*Ae26*), *P.* *dioica* (*Ae28*), *C.* *sativum* (*Ae29*), *P.* *boldus* (*Ae32*), *L.* *nobilis* (*Ae33*), and *O.* *quixos* (*Ae36*)

When comparing results, 11 of the 15 active essential oils (73%) coincided with their MIC values, using both methods; 4 of the 15 (27%) have a different value as observed in Fig. 1. The difference in MIC values was only with one concentration. This is probably because the value was in the limit of two successive concentrations. Additionally, the serial dilutions and the adherence of the essential oils to the polypropylene pipette points could also affect results.

In Yates correction, a Chi-Square value of 6.05 was obtained. Meanwhile, the theoretical value (critical value) with 5 freedom degrees was 11.07, with a *p* value 0.63. For Montecarlo simulation the *p* value was  > 0.7, and for Fisher´s exact test *p*  > 0.05. With these results, did not exist enough criteria to reject the null hypothesis. Thus, it is assumed that the applied methodologies and the MIC values are independent, which confirms that there does not exist a value of MIC that has a preference for the applied methods.

The dilution in agar and broth dilution methods allow to compare and determine the MIC of the evaluated essential oils. Small variations in MIC values could be attributed to emulsion stability and the microtechnique limitations [8].

The microdilution modified method for the evaluation of essential oils activity presented in this study is an effective procedure for determining MIC values of these kind of compounds. The constant homogenization, the use of microtubes, and the use of film paper over the microplate wells are important factors for the success of this methodology as it is shown when comparing the sensitivity and specificity of both methods. However, the proposed microdilution method permits savings in resources and time, principally due to the possibility to evaluate multiple essential oils in the same microplate.

### Limitations

The presented method is still semi quantitative and does not allow the determination of neither the % of inhibition nor the IC_50_ or IC_90_. Values at limits of the different ranges, for example between 0.24 and 0.48% v/v are critical, because of the fact that minimal variations of MIC will transfer to the next group. Quantification of activity by tetrazolium salts are proposed for a next stage.

## Supplementary Information

**Additional file 1: Table 1.** Essential oils of therapeutic grade tested and references of their activity or no activity against *E. coli* strains. **Table 2.** Essential oils of medicinal plants from local markets, and references of their activity or no activity against *E. coli* strains

**Additional file 2: Figure 1.** Comparison of the antimicrobial activity of the essential oils at a MIC ≤0.48% (v/v) for the micro and macro dilution methods of essential oils.

## Data Availability

The datasets used and/or analyzed during the current study are available from the corresponding author on reasonable request.

## Abbreviations

*E.**coli*: *Escherichia* *coli*
CFU: Colony forming units
MIC: Minimum inhibitory concentration
MHA: Mueller Hinton Agar
ATCC: American Type Culture Collection
*E.**citriodora*: *Eucalyptus* *citriodora*
*Z.**officinale*: *Zingiber* *officinale*
*S.**aromaticum*: *Syzygium* *aromaticum*
*C.**cassia*: *Cinnamomum* *cassia*
*L.**angustifolia*: *Lavandula* *angustifolia*
*C.**flexuous*: *Cymbopogon* *flexuous*
*M.**spicata*: *Mentha* *spicata*
*M.**alternifolia*: *Melaleuca* *alternifolia*
*C.**cyminum*: *Cuminum* *cyminum*
*P.**dioica*: *Pimenta* *dioica*
*C.**sativum*: *Coriandrum* *sativum*
*P.**boldus*: *Peumus* *boldus*
*L.**nobilis*: *Laurus* *nobilis*
*O.**quixos*: *Ocotea* *quixos*
