# Correction to: Validation of a method of broth microdilution for the determination of antibacterial activity of essential oils

**DOI:** 10.1186/s13104-022-05915-6

**Published:** 2022-02-14

**Authors:** David Vanegas, Andrea Abril-Novillo, Aleksandr Khachatryan, Lourdes Jerves-Andrade, Eugenia Peñaherrera, Nancy Cuzco, Isabel Wilches, Jessica Calle, Fabián León-Tamariz

**Affiliations:** Department of Biosciences, Group of Medicinal Plants and Natural Products, Faculty of Chemistry, School of Biochemistry and Pharmacy, Universidad de Cuenca, Cuenca, Ecuador

## Correction to: BMC Research Notes (2021) 14:439 https://doi.org/10.1186/s13104-021-05838-8

Following the publication of the original article [1] we were informed that the authors’ given and family names had unfortunately been interchanged.

The author names have been corrected in the author list of this Correction and updated in the original article.
