# Supplementary material for: Validation of a method of broth microdilution for the determination of antibacterial activity of essential oils
Source: BMC Res Notes. 2021 Dec 2;14:439. doi: 10.1186/s13104-021-05838-8 (PMC8638534; doi:10.1186/s13104-021-05838-8)
Supplement: Supplementary file 1 — Additional file 1: Table 1. Essential oils of therapeutic grade tested and references of their activity or no activity against E. coli strains. Table 2. Essential oils of medicinal plants from local markets, and references of their activity or no activity against E. coli strains [file 13104_2021_5838_MOESM1_ESM.docx]

**Table 1** Essential oils of therapeutic grade tested and references of their activity or no activity against *E. coli* strains.

| **Essential oil** | **Common name** | **Part used ^∆^** | **Reported antibacterial activity MIC*** | **Reference** |
| --- | --- | --- | --- | --- |
| *Eucalyptus globulus* | Blue gum | LT | >0.5%v/v | (10) |
| *Syzygium aromaticum* | Clove | BD | 0,24%v/v | (10) |
| *Cinnamomum cassi* | Chinese cinnamon | W | 0,1%v/v | (11) |
| *Boswellia serrata* | Sallaki | R | 9, 25%v/v | (12) |
| *Lavandula angustifolia* | Lavender | FL | 1,07%v/v | (13) |
| *Cymbopogon flexuous* | Cochin grass | LT | >0.08%v/v | (14) |
| *Mentha x piperita* | Peppermint | LT | 0,004%v/v | (15) |
| *Rosmarinus officinalis* | Rosemary | LT | 0,014%v/v | (4) |
| *Mentha spicata* | Spearmint | LT | 0,04%v/v | (15) |
| *Melaleuca alternifolia* | Tea tree | LT | >1%v/v | (16) |
| *Citrus racemose* | Grapefruit | P | 0,63%v/v | (17) |
| *Citrus limonum* | Lemon | P | 1%v/v | (18) |
| *Citrus aurantifolia* | Key lime | P | 1%v/v | (19) |
| *Citrus* *sinensis* | Sweet orange | P | 0,08%v/v | (20) |
| *Pogostemon cablin* | Patchouli | NN | 0,05%v/v | (16) |
| *Salvia sclarea* | Clary sage | NN | >53,2%v/v | (21) |
| *Citrus bergamia* | Bergamot orange | NN | 1%v/v | (18) |

^∆=^BD, bud; FL, ﬂower; LT, leaves; P, peel; NN not named; R, resin; W, wood.

* The range of activity/no-activity were determined based on reported MIC and the cytotoxicity of essential oils.

**Table 2** Essential oils of medicinal plants from local markets, and references of their activity or no activity against *E. coli* strains

| **Plants** | **Common name** | **Plant part** | **Reported antibacterial activity MIC*** | **Reference** |
| --- | --- | --- | --- | --- |
| *Zingiber officinale* | Ginger | RH | >2%v/v | (19) |
| *Foeniculum vulgare* | Fennel | S | 0,007%v/v | (4) |
| *Pimpinella anisum* | Anise | S | 0,10%v/v | (22) |
| *Coriandrum sativum* | Coriander | S | 0,07%v/v | (23) |
| *Curcuma longa* | Turmeric | RH | 48,02%v/v | (24) |
| *Piper nigrum* | Black pepper | FR | >2%v/v | (19) |
| *Peumus boldus* | Boldo | LT (dry) | 0,003%v/ | (4) |
| *Laurus nobilis* | Baby laurel | LT (dry) | 1,25%v/v | (25) |
| *Petroselinum crispum* | Parsley | S | 1,08%v/v | (3) |
| *Elettaria cardamomum* | Green cardamom | S | 1%v/v | (26) |
| *Ocotea quixos* | Ishpingo | S | 1,68%v/v | (27) |
| *Eucalyptus citriodora* | Eucalyptus | LT | 0,2%v/v | (21) |
| *Salvia microphylla* | Baby sage | LT | >53,2%v/v | (28) |
| *Cuminum cyminum* | Cumin | S | 0,001%v/v | (29) |
| *Illicium verum* | Star anise | S | 0,001%v/v | (30) |
| *Pimenta dioica* | Allspice | FR | 0,19%v/v | (22) |

^∆=^FR, fruit; L T, leaves; RH, rhizome; S, seed.

* The range of activity/no-activity were determined based on reported MIC and the cytotoxicity of essential oils.
