# Supplementary figures and images for: Validation of a method of broth microdilution for the determination of antibacterial activity of essential oils
Source: BMC Res Notes. 2021 Dec 2;14:439. doi: 10.1186/s13104-021-05838-8 (PMC8638534; doi:10.1186/s13104-021-05838-8)

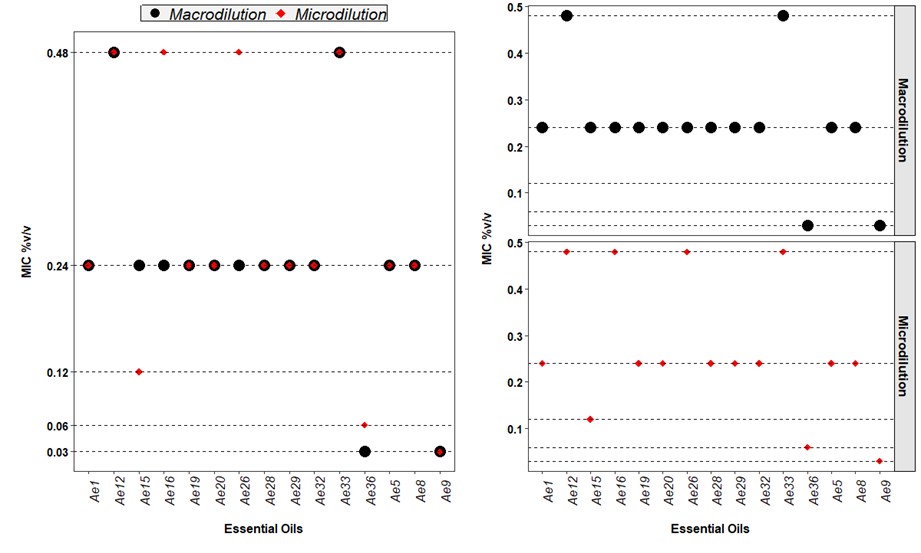

Supplement: Supplementary file 2 — Additional file 2: Figure 1. Comparison of the antimicrobial activity of the essential oils at a MIC ≤0.48% (v/v) for the micro and macro dilution methods of essential oils. [file 13104_2021_5838_MOESM2_ESM.docx]
